# Supplementary material for: Complex‐centric proteome profiling by SEC‐SWATH‐MS
Source: Mol Syst Biol. 2019 Jan 14;15(1):e8438. doi: 10.15252/msb.20188438 (PMC6346213; doi:10.15252/msb.20188438)
Supplement: Supplementary file 6 — Dataset EV5 [file MSB-15-e8438-s006.zip › feature_plots_corum/633.pdf]

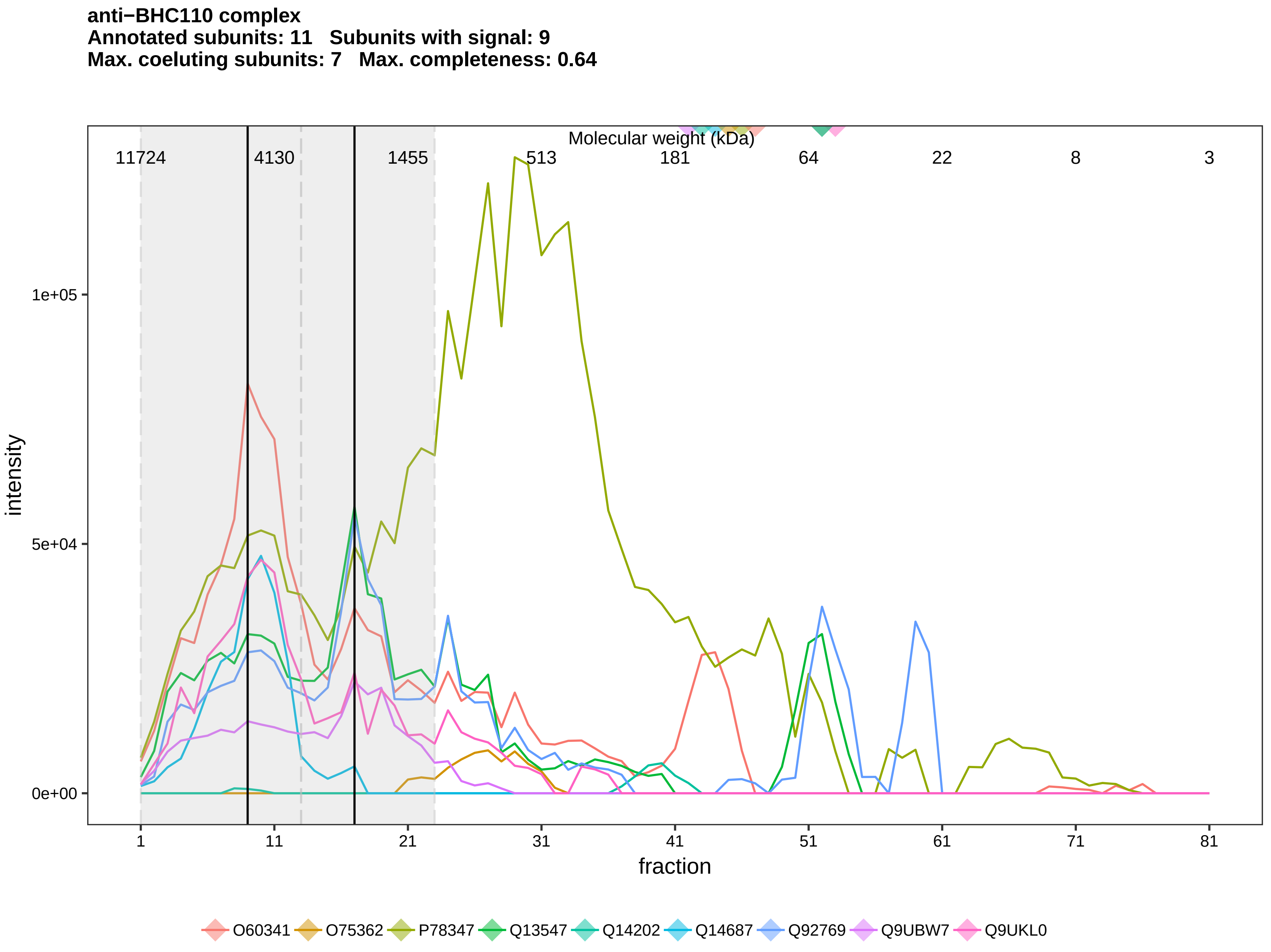
anti−BHC110 complex
Annotated subunits: 11 Subunits with signal: 9
Max. coeluting subunits: 7 Max. completeness: 0.64
Molecular weight (kDa)
11724
4130
1455
513
181
64
22
8
3
1e+05
5e+04
0e+00
intensity
1
11
21
31
41
51
61
71
81
fraction
O60341
O75362
P78347
Q13547
Q14202
Q14687
Q92769
Q9UBW7
Q9UKL0
